# Teledentistry Applied to Health and Education Outcomes: Evidence Gap Map

**DOI:** 10.2196/60590

**Published:** 2024-11-27

**Authors:** Júlia Nascimento da Silva Mulder, Marcelo Ramos Pinto, Isabelle Aníbal, Ana Paula Dornellas, Deise Garrido, Camila Huanca, Ana Estela Haddad, Carmen Verônica Mendes Abdala

**Affiliations:** 1 Department of Orthodontics and Pediatric Dentistry University of São Paulo - School of Dentistry São Paulo Brazil; 2 Latin American and Caribbean Center on Health Sciences Information São Paulo Brazil

**Keywords:** teledentistry, systematic review, dental education, dentistry, telemedicine, research design, health information technologies, mobile phone

## Abstract

**Background:**

Teledentistry is a field of activities that comprises information and communication technologies (ICTs) applied to dentistry, including the exchange of clinical information, patient care, and the use of educational strategies across remote distances. Its use has grown progressively over the past decades—intensified by the COVID-19 pandemic—and has been improving the provision of dental services and educational strategies ever since.

**Objective:**

This evidence gap map (EGM) study aims to present a collection of systematic reviews (SRs) with meta-analyses to answer the question “What are the applications of teledentistry in dental services and dental education?” by identifying gaps and current evidence on the improvement of health care and education.

**Methods:**

The EGM methodology has been developed by the Latin American and Caribbean Center on Health Sciences Information and is based on the concept created by the International Initiative for Impact Evaluation. Embase, PubMed, and Virtual Health Library databases were used for the literature research, using terms for teledentistry associated with eHealth, dental education, and oral health care. The data obtained from the included studies were then characterized in a Microsoft Excel spreadsheet, with a matrix containing 8 intervention groups (combined interventions, e-learning and tele-education, teleconsultation and teleservice, telemonitoring, telediagnosis, telescreening, ICTs, and artificial intelligence) and 8 outcome groups (diagnosis accuracy, education and professional training, user behavior, clinical practice, patient-centered outcomes, clinical outcomes, health services management, and access to health services). The quality of the studies was assessed using AMSTAR2 (A Measurement Tool to Assess Systematic Reviews). The visual analytics platform Tableau (Salesforce) was used to graphically display the confidence level, number of reviews, health outcomes, and intervention effects.

**Results:**

The confidence level obtained by the criteria applied was high for 28% (19/68) of the studies, moderate for 6% (4/68), low for 15% (10/68), and critically low for 51% (35/68). Among the interventions, the ICT group stood out with 182 (36.8%) out of 494 associations, followed by interventions with e-learning and tele-education (n=96, 19.4% of associations), telediagnosis (n=67, 13.6%), and combined interventions (n=53, 10.7%). Most of the outcomes were aimed at education and professional training (97/494, 19.6% of associations), patient-centered outcomes (74/494, 15%), and health services management (60/494, 12.1%).

**Conclusions:**

This EGM presents an overview of the contributions of teledentistry in patient care, health services, clinical practice, and education. The study results may help guide future research and policy decisions and serve as a convenient virtual tool for accessing valuable evidence-based information on teledentistry.

## Introduction

### Background

The World Health Organization defines telehealth as the exchange of health information mediated by information and communication technologies (ICTs) for the purposes of health care, education, research, and management of health care services or networks [1]. Although telehealth was already adopted in many countries, its use and applications experienced exponential growth during the COVID-19 pandemic, as health services worldwide were partially or completely interrupted [2-4]. The concept of telehealth applied to dentistry is labeled as teledentistry, and through the use of ICTs, it has been qualifying the provision of dental services directed at improving patient care as well as professional education and training [5]. However, despite teledentistry being a current and global reality, with a great range of reported applications and experiences, evidence gaps for the consolidation of educational and health strategies still exist [6-8].

Considering the importance of dentistry at both individual and population levels, it is logical and advisable that teledentistry is incorporated into health care and, consequently, into teaching [2,9,10]. With the expansion of care integrated into the teaching-service axis, teledentistry favors the dynamics of dental care systems and training, allowing new educational opportunities [10,11]. However, for teledentistry to fulfill its maximum potential within teaching and health care, legal support and the involvement of all policy makers are necessary to enable the optimization and expansion of services, care, and patients’ referrals to specialists as well as to assist students through interprofessional interaction, experience, tool development, and remote supervision of patients [7,11-14].

The evidence gap map (EGM) methodology aims to identify current evidence and knowledge gaps on a given subject of study. The given results provide guidance for new research as well as public policy making [15]. The EGM methodology was created by the International Initiative for Impact Evaluation—a global initiative established in 2008 that seeks to develop scientific evidence for transformation effects on the lives of populations in low- and middle-income countries. The Latin American and Caribbean Center for Health Sciences Information of the Pan American Health Organization (BIREME/OPAS) uses the International Initiative for Impact Evaluation strategy to support investments directed at the production of more evidence or the synthesis of existing evidence [15-17].

### This Study

This EGM presents an overview of the evidence on the effects of teledentistry for a variety of health and education outcomes, such as diagnosis accuracy, education and professional training, user behavior, clinical practice, patient-centered outcomes, clinical outcomes, health services management, and access to health services [15-17].

## Methods

### Overview

This study was based on the application of the EGM methodology, which consists of graphically representing the characteristics and findings of the evidence analyzed in systematic reviews (SRs), associating interventions with the outcomes analyzed in the reviews, and linking them with the reported effects of the interventions (with the population and focus countries of the primary studies included in the reviews).

On the map, associations are represented using bubbles of different colors, which represent the confidence level of the reported evidence and the effects of the intervention for each different health outcome. The bubble size is equivalent to the number of studies that analyzed each association. All bubbles lead to a list of study titles, with a link to the full text.

SRs—with or without meta-analysis—that could answer the research question “What are the applications of teledentistry in dental services and dental education?” were eligible for inclusion in the EGM.

For the development of this EGM, five different phases respecting the proposed methodology were applied: literature bibliographic search (phase 1), reference management (phase 2), study characterization (phase 3), quality assessment and report (phase 4), and infographics (phase 5).

### Search Strategy

A bibliographic search (EGM phase 1) was conducted using Medical Subject Headings and DeCS terms in English, Portuguese, and Spanish within the scope of telehealth, dentistry, and teledentistry. This search was applied to PubMed (MEDLINE) and 2 additional databases—Virtual Health Library (LILACS, BBO, WHOLIS, and Pan American Health Organization IRIS) and Embase (Elsevier)—excluding results already obtained from PubMed. The full list of terms used in the search can be found in Multimedia Appendix 1. A filter for SRs and meta-analysis on teledentistry was applied to the consulted databases, and the results respected the following PICO strategy:

- P=patients, students, and health professionals
- I=teledentistry
- C=conventional dentistry
- O=health and education

A team of calibrated experts from the Telehealth and Teledentistry Center FOUSP-SAITE (NuTes-FOUSP, University of São Paulo, SP, Brazil), along with researchers and librarians from BIREME/OPAS, was consulted to cross-review the search strategy before establishing the inclusion and exclusion criteria. No disagreement was found between the examiners during this phase. The detailed search strategy can be found in Multimedia Appendix 1.

It is important to point out that the specific term “evidence gap map” (or “evidence map”) is not yet included in the Medical Subject Headings and DeCS list till the date of submission of this paper. Furthermore, the expression “research design” was used as the most similar research term.

The term ICT was kept as the main reference, as it was once a part of the majority of articles and is quoted this way in the EGM. However, the researchers acknowledge the existence of the term “digital information and communication technologies,” which is being used more frequently to differentiate the forms of technology applied in dentistry (nondigital and digital).

### Inclusion Criteria

The inclusion criteria for the studies were as follows: clinical applications of teledentistry; educational applications of teledentistry; applications directly in services; a variety of ICTs with applications in dentistry; SRs and meta-analysis; and studies written in Portuguese, English, or Spanish.

### Exclusion Criteria

The exclusion criteria for the studies were as follows: ICTs with no clinical or educational applications, clinical approaches without the use of ICT, clinical studies based on strictly laboratory or laboratory research in the experimental phase, research outside the scope of teledentistry, and studies that were not SRs or meta-analyses.

### Data Extraction and Risk of Bias Assessment

Examination of search results was performed through Rayyan (Rayyan Company) reference management software (an artificial intelligence [AI]–assisted systematic literature review tool, from Rayyan Company) by 2 independent and blinded literature reviewers (phase 2) [18]. In case of disagreement among researchers regarding including or excluding an article (based on the inclusion and exclusion criteria), a third researcher of the group, who was still “blinded,” made the final decision. Then, the duplicate articles were removed. This platform manages the risk of bias related to influence between individuals and offers a reasonable interface, with a dashboard that informs the overall status about inclusion decisions (undecided, maybe, included, excluded, or conflict) as well as possible duplicates (unresolved, resolved, not duplicates, or deleted).

After full-text reading, for the further evaluation and confidence level classification of studies (equivalent to EGM’s phase 4), the AMSTAR2 (A Measurement Tool to Assess Systematic Reviews) checklist was used. This was also established by 2 independent reviewers as a strategy for risk of bias management. AMSTAR2 is aimed at evidence-based health care and establishes 16 questions for the appraisal of SRs to be scored as “yes,” “partially yes,” or “no” depending on the question and the detailed data retrieved from the review. The questions cover various aspects, including adherence to protocol statements, inclusion criteria, search strategy methods, duplication during study selection and data extraction, justification for excluded studies, detailed descriptions of included studies, risk of bias assessment, funding information, statistical analysis, management of heterogeneity, and conflicts of interest, among others. From these 16 questions, 7 (%) are considered “critical” for quality evaluation. If a study presents no critical weaknesses and up to 1 noncritical weakness, it can be considered a “high” quality review; for studies with no critical weakness but >1 noncritical weakness, it can be considered a “moderate” quality review; for studies with 1 critical weakness (with or without other noncritical weaknesses), the study is to be labeled as a “low” quality review; and studies with >1 critical weakness are labeled “critically low” quality review [19,20]. The list of the included studies can be found in Table 1. The numbered references are presented in Multimedia Appendix 2 [21-88], and the list of the excluded studies after full-text appraisal are presented in Multimedia Appendix 3.

**Table 1:** List of studies included in the teledentistry evidence gap map. For details about the included studies, please refer to Multimedia Appendix 2.

Reference number of the included study, quoted in Multimedia Appendix 2	Focus countries	Publication year	AMSTAR2^a^ confidence level	Main study focus
1	Not specified	2023	Low	Management of pain and anxiety
2	Europe, the United States, Australia, Japan, and India	2018	Critically low	Teledentistry evaluation
3	South Korea, Egypt, Australia, and the United Kingdom	2023	Low	Education and learning
4	Not specified	2021	Critically low	Management of pain and anxiety
5	Not specified	2022	Critically low	Management of pain and anxiety
6	The United States, the United Kingdom, Brazil, Portugal, Australia, and Germany	2018	Critically low	Teledentistry evaluation
7	North America, Latin America, Europe, and Asia	2015	Critically low	Diagnosis
8	India, Brazil, and Malaysia	2023	High	Diagnosis
9	Not specified	2019	Critically low	Education and learning
10	Not specified	2022	Critically low	Education and learning
11	Germany, Saudi Arabia, and the United States	2023	High	Patient care
12	China, Japan, Finland, Portugal, the United States, Germany, and Israel	2023	Low	Virtual reality and artificial intelligence
13	Saudi Arabia, India, Brazil, Australia, the United States, France, Malaysia, and South Korea	2020	Critically low	Dental practice and training
14	The United States, Europe, and Asia	2019	Critically low	Management of pain and anxiety
15	Pakistan, Colombia, India, and Saudi Arabia	2022	Critically low	Teledentistry during the COVID-19 pandemic
16	Iran, Brazil, the United States, Saudi Arabia, India, and Australia	2022	High	Patient care
17	Saudi Arabia, Australia, the United States, Iran, and India	2023	High	Patient care
18	Australia, France, and Germany	2020	Critically low	Access to care and health systems
19	Greece, Philippines, the United States, China, India, South Korea, Germany, Pakistan, and Vietnam	2023	High	Diagnosis
20	Portugal, the United Kingdom, the Netherlands, Turkey, Australia, the United States, and Brazil	2016	Critically low	Diagnosis
21	The United States, India, Italy, Australia, Canada, Iran, China, and Turkey	2020	Critically low	Management of pain and anxiety
22	The United Kingdom, the United States, North America, and Malaysia	2020	Critically low	Education and learning
23	Not specified	2023	High	Education and learning
24	The United States, the United Kingdom, Brazil, Germany, Australia, Greece, Iran, and Saudi Arabia	2021	Critically low	Education and learning
25	The United States and Spain	2023	Low	Education and learning
26	Iraq, Saudi Arabia, the United States, Italy, Peru, Pakistan, India, and China	2019	Critically low	Patient care
27	Not specified	2023	Moderate	Dental monitoring
28	Brazil, Canada, China, Costa Rica, France, Nepal, Peru, Serbia, and the United States	2016	Moderate	Education and learning
29	Not specified	2023	Low	Education and learning and dental practice and training
30	The United States, Germany, and the United Kingdom	2019	Critically low	Education and learning
31	Iran, India, Greece, Syria, Turkey, and Jordan	2023	High	Patient care
32	The United Kingdom, the United States, Australia, the Netherlands, Brazil, Thailand, Spain, France, Hong Kong, Germany, China, India, Serbia, Iran, Japan, Hungary, Sweden, South Korea, and Saudi Arabia	2022	Critically low	Education and learning
33	India, Iran, Turkey, and China	2023	High	Management of pain and anxiety
34	Thailand, Mexico, Iran, Saudi Arabia, Egypt, India, and Syria	2020	Moderate	Management of pain and anxiety
35	The Netherlands, India, the United States, Italy, and China	2021	Critically low	Dental monitoring
36	Not specified	2023	High	Teledentistry during the COVID-19 pandemic and education and learning
37	The United States, France, Japan, Brazil, China, Australia, the United Kingdom, the Netherlands, and Thailand	2023	High	Education and learning
38	Bahrain, Australia, the United Kingdom, Africa, Finland, Canada, Spain, United Arab Emirates, and the United States	2023	Low	Education and learning and patient care
39	Not specified	2023	High	Dental monitoring
40	Not specified	2020	Critically low	Management of pain and anxiety
41	Not specified	2022	Critically low	Patient care
42	Saudi Arabia, the Netherlands, Germany, Romania, Brazil, Italy, Turkey, Bulgaria, Canada, and Israel	2022	Low	Dental monitoring
43	The United States, Germany, the United Kingdom, Australia, Spain, Sweden, Canada, Brazil, China, the Netherlands, Switzerland, Belgium, Finland, Thailand, and Taiwan	2014	High	Education and learning and patient care
44	The United Kingdom, Italy, India, Iran, Saudi Arabia, Peru, Brazil, and Malaysia	2023	High	Patient care
45	Canada, the United Kingdom, Finland, Spain, India, the United States, Italy, and Australia	2017	Low	Patient care
46	The United States, Israel, China, Italy, Jordan, and Austria	2020	Critically low	Dental practice and training and teledentistry during the COVID-19 pandemic
47	India	2022	Critically low	Access to care and health systems
48	The United Kingdom, the United States, Italy, Australia, India, and China	2022	High	Dental practice and training
49	Iran, India, Italy, the Netherlands, Australia, England, Brazil, Niger, and Syria	2022	Critically low	Patient care
50	Not specified	2021	Critically low	Dental practice and training
51	Not specified	2021	Critically low	Dental practice and training
52	Not specified	2023	High	Dental monitoring
53	Iraq, Saudi Arabia, Germany, Portugal, the United States, Italy, Belgium, Pakistan, India, China, Brazil, and the Netherlands	2021	Low	Oral health promotion
54	Brazil, India, and the United Kingdom	2020	Critically low	Diagnosis
55	India, Brazil, Malaysia, the United States, and Italy	2023	High	Diagnosis
56	Not specified	2013	Critically low	Dental practice and training
57	The United States, Europe, and middle-income countries	2013	Critically low	Teledentistry evaluation
58	Spain and Sweden	2023	Moderate	Diagnosis and dental monitoring
59	Not specified	2023	Low	Dental practice and training
60	The United States, Italy, Taiwan, and the Netherlands	2022	High	Dental monitoring
61	The United Kingdom, Saudi Arabia, Germany, England, the Netherlands, Turkey, and Italy	2022	High	Patient care
62	Brazil, the United States, Greece, Germany, Portugal, and the United Kingdom	2021	High	Teledentistry during the COVID-19 pandemic
63	The United States	2022	Critically low	Patient care
64	Not specified	2020	Critically low	Management of pain and anxiety
65	The United States, the United Kingdom, Serbia, Brazil, Sweden, Spain, Taiwan, Portugal, Germany, Australia, Finland, Canada, and Austria	2018	Critically low	Patient care
66	Syria, Iran, the United States, Malaysia, Finland, India, the United Kingdom, Türkiye, Canada, and Australia	2021	Critically low	Virtual reality and artificial intelligence
67	Iran and England	2021	Critically low	Education and learning and patient care
68	Not specified	2022	Critically low	Virtual reality and artificial intelligence

^a^AMSTAR2: A Measurement Tool to Assess Systematic Reviews.

Regarding data safety and protection protocols, the research team only worked with digital information and files (and not physical or printed documents). All digital files were stored on dedicated servers at the University of São Paulo and BIREME, with access restricted to researchers through a password.

### Ethical Considerations

As EGM studies apply no direct interventions on individuals or animals, there was no need for study submission to the ethics committee. No sensitive individual (general or health) information was directly generated by the researchers. The use of AI tools was indirect and restricted to the ones already embodied in the platforms or applications used by the researchers; there was no direct application of deep learning and natural language processing (chatbots, generative pretrained transformers, or similar engines) to compose the writing or the revision of this paper.

### Data Synthesis and Characterization

A team of 7 dentists who were also researchers from the Telehealth and Teledentistry Center FOUSP-SAITE (NuTes-FOUSP, University of São Paulo, São Paulo, Brazil) worked in collaboration with a team of 3 researchers from the Latin American and Caribbean Center for Health Sciences Information (BIREME/OPAS/World Health Organization) to analyze the data of the included studies.

After full-text analysis, a matrix on Microsoft Excel was developed based on the evidence map methodology to organize the data extracted from the included studies to summarize and measure the results found for all the teledentistry actions mentioned and to create graphic elements to be inserted later on in the interactive map (phase 3). For each row of the selected studies, the detailed matrix included the following: title, intervention group, the individual intervention to be measured, the outcome group, the individual outcome that the intervention seeks to achieve, the effect obtained by the association of both individual intervention and outcome, the focus population of the intervention, the database used to obtain the full text and the ID, the confidence level obtained through the AMSTAR2 criteria, the type of review, the study design of the primary studies included in the review, the review design, the focus and publication countries, the publication year, and a link to the full text. A tab with the possible results for the type of review, review design, study design, confidence level, effects, and populations was created for the automatic count, while interventions and outcomes were grouped and coded before automatic counting. The specific data for each study was counted using the filters available in Microsoft Excel. Table 2 presents the list of outcome groups and the number of associations found for each individual outcome, while Table 3 presents the list of intervention groups and number of associations found for each individual intervention.

The associations and results found were then counted through the use of Microsoft Excel’s filters (following the manual check) for further creation and publication of the interactive evidence map using the Tableau platform in an area created for BIREME (Tableau Public [89]), graphically demonstrating the panorama of consolidated evidence and the gaps identified in the selected studies through the inclusion criteria and entered into the characterization matrix (phase 5, all available via reference 21 in the reference list).

The interactive map is divided into three main sections: (1) general, (2) confidence level, and (3) intervention effect. In the first one, same-colored bubbles are presented to illustrate the volume of SRs published for a certain association (outcome vs intervention crossing). For confidence level, these bubbles were either green, yellow, red, or blue, indicating associations belonging to reviews that have achieved high, moderate, low, or critically low confidence levels, respectively, on AMSTAR2. Regarding the intervention effect section, associations will be illustrated by dark green (for positive effects), light green (for potential positive), orange (potential negative), red (negative), pink (inconclusive), light blue (no effect), or gray (no reported effect). By directing the cursor over the bubble in any section, a pop-up appears, presenting the number of reviews and publication countries that have analyzed this particular association effect. A list of the included studies can be found right below the map to provide open access to the reviews. The map also presents a summary of the main findings and information about the authors and collaborators [89,90].

Table 2 presents the relationship between the outcome groups and individual outcomes, followed by the prevalence of associations found for each of these.

Table 3 presents the relationship between the intervention groups and individual interventions, followed by the prevalence of associations found for each of these.

**Table 2:** List of outcome groups and the number of associations found for each individual outcome.

Outcome groups and outcomes		Associations found, n	Associations by outcome group, n		Equivalence to the total number of associations (%)	
**Access to health services**				21		4
	Convenience	2				
	Service quality	10				
	Reduction of unnecessary traveling	1				
	Wait reduction	4				
	Line reduction	2				
	Surgery access	2				
**Diagnosis accuracy**				44		9
	Carious lesions	10				
	Enamel defects	2				
	Oral conditions	3				
	Trauma	2				
	Diagnosis in general	8				
	Erosion	2				
	Fluorosis	2				
	Cancer	7				
	Potentially malignant oral lesions	7				
	Urgency detection	1				
**Clinical outcomes**				66		13
	Gingival inflammation reduction	10				
	Plaque index reduction	11				
	Probing depth reduction	2				
	Probing bleeding reduction	2				
	Oral disease prevention	19				
	Cross infection Prevention	1				
	Clinical outcomes in general	7				
	Prognosis improvement	13				
	Orthodontic relapse	1				
**Education and professional training**				97		20
	Learning	26				
	Assessment performance	6				
	Professional training	21				
	Clinical skills improvement	24				
	Increase in student’s confidence	5				
	Student’s stress management	4				
	Pedagogical model	6				
	Educational path	4				
	Student’s convenience	1				
**Health services management**				60		12
	Health care extension	21				
	Patient monitoring	5				
	Dental emergencies	2				
	Time optimization	18				
	Regulation and health care systems	3				
	Cost reduction	7				
	Decrease on appointments	4				
**Patient-centered outcomes**				74		15
	Distress relief	1				
	Health behavior change	14				
	Psychological impact relief	1				
	Health education	16				
	Safety	2				
	Behavior along treatment	6				
	Anxiety relief	13				
	Pain perception relief	12				
	Change in hygiene habits	9				
**Clinical practice**				75		15
	Clinical training	9				
	Decision-making	13				
	Communication among professionals	4				
	Professional and patient communication	2				
	Procedure planning	4				
	Applied to restorative dentistry	2				
	Applied to radiology	2				
	Applied to orthodontics	14				
	Applied to oral surgery	5				
	Digital planning	8				
	Human relations	5				
	Control of events and complications	7				
**User behavior**				57		12
	Adherence to ICT^a^	4				
	Treatment adherence	10				
	ICT acceptability	9				
	ICT reliability	1				
	ICT perception	8				
	ICT satisfaction	11				
	Tool use	11				
	Integration with other ICT	2				
	Treatment fit	1				
71 outcomes organized into 8 groups		494	494		100	

^a^ICT: information and communication technology.

**Table 3:** List of intervention groups and number of associations found for each individual intervention.

Intervention groups and interventions			Associations found, n		Associations by intervention group, n			Equivalence to the total number of associations (%)	
**Combined interventions**						53			11
	Tele-education and virtual reality	11							
	Telescreening and telediagnosis	15							
	Multiple approaches in teledentistry	25							
	Surgical supplementation	2							
**Teleconsultation and teleservice**						36			7
	Telecare	8							
	Personalized teleservice through videoconferencing	1							
	Teleconsultation	18							
	Tele-interconsultation	9							
**Telediagnosis**						67			14
	Telediagnosis (through smartphone)	16							
	Telediagnosis (through clinical photography)	15							
	Telediagnosis (through digital cameras)	6							
	Telediagnosis in general	30							
**e-Learning and tele-education**						96			19
	e-Learning	19							
	Digital approaches in dental education	27							
	Virtual learning environments	13							
	Tele-education (hybrid teaching)	16							
	Teleducação (emergency basis)	7							
	Tele-education (through apps and SMS)	2							
	Tele-education (through videos)	4							
	Tele-education (through MOOCs^a^)	2							
	Telementoring	6							
**ICTs** ^b^						182			37
	Digital distraction and virtual reality	24							
	Technology-based interventions	54							
	eHealth technologies	10							
	Educational games (through app)	11							
	Educational games (through social media)	2							
	mHealth^c^	32							
	Social media	21							
	Virtual reality	15							
	Videos (YouTube and other media)	2							
	Digital information and communication technologies	10							
	Relaxing music	1							
**Telemonitoring**						44			9
	Telemonitoring in general	27							
	Telemonitoring through app	12							
	Telemonitoring through email	2							
	Telemonitoring through voice message	1							
	Telemonitoring through WhatsApp	2							
**Telescreening**						7			1
	Telescreening	5							
	Telescreening through smartphone	2							
**Artificial intelligence**						9			2
	Diagnosis via a professional camera picture	1							
	Diagnosis via an intraoral camera picture	1							
	Diagnosis via a smartphone picture	1							
	Artificial intelligence	6							
43 interventions organized into 8 groups			494		494			100	

^a^MOOC: Massive Open Online Course.

^b^ICT: information and communication technology.

^c^mHealth: mobile health.

## Results

### Overview

The comprehensive bibliographic search, conducted in December 2023 to ensure the most up-to-date inventory of articles, retrieved a total of 504 records from the PubMed, Virtual Health Library, and Embase databases (Multimedia Appendix 2). After retrieving the records on Rayyan, we excluded duplicates and screened the remaining papers based on inclusion and exclusion criteria. Full texts of 118 studies were read. Following this, 50 studies were excluded (based on the same criteria) by 2 independent reviewers.

**Figure 1:**
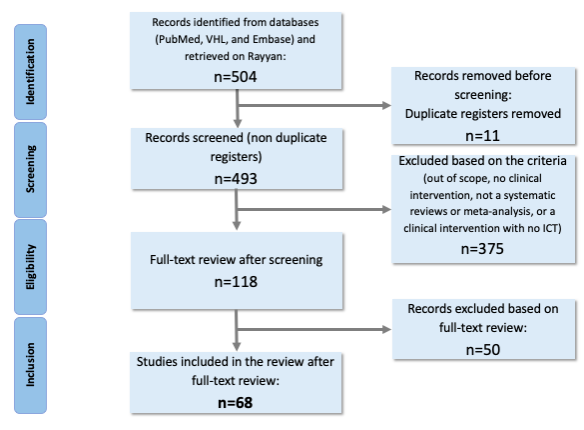
PRISMA (Preferred Reporting Items for Systematic Reviews and Meta-Analyses) diagram based on the search strategy and inclusion and exclusion criteria.

The excluded studies and reasons for exclusion can be found in Multimedia Appendix 3. After excluding duplicates from different sources and analyzing titles, abstracts, and full texts, a total of 68 studies were included in the teledentistry EGM. Although the PRISMA (Preferred Reporting Items for Systematic Reviews and Meta-Analyses) protocol is recommended for SRs—and the EGM concept aims for a complementary overall design direction—it was used as a methodological step to enforce the quality of the SRs selected, followed by the application of inclusion and exclusion criteria and full-text readings to check for the eligibility of the studies (Figure 1).

### Main Results

The map presents an overview of the evidence on the applicability and effects of teledentistry for health and education outcomes. From a comprehensive bibliographic search, 68 reviews were included in the map (SRs: n=51, 75%; SRs with meta-analysis: n=16, 24%; and meta-analysis: n=1, 1%). Table 4 presents the main positive and potentially positive associations found in the high confidence–level reviews included in the map. Table 4 presents the reviews with a high level of confidence included in the map (following the AMSTAR2 criteria) along with focus populations and main positive and potentially positive effects.

In total, 494 associations were found between the combinations of teledentistry+health and teledentistry+education outcomes. The effect reported by the review study was included for each intervention-outcome association. For most associations, the positive or potentially positive effect of teledentistry was reported (434/494, 87.8% associations total). For other associations, an inconclusive or no effect was reported (39/494, 7.9% associations total). The negative or potentially negative effect of teledentistry was reported for only 0.1% (5/494) of the associations. The effect was not reported for 3.2% (16/494) of the associations.

**Figure 2:**
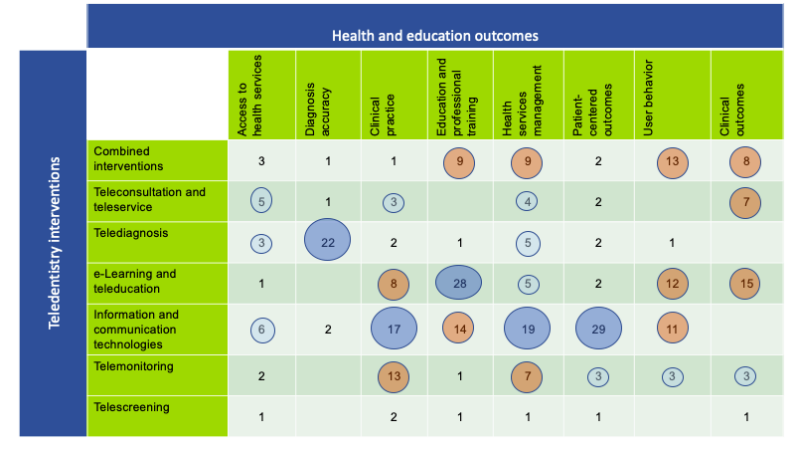
Partial visual representation of the evidence gap map, with the main groups for both teledentistry interventions and health and education outcomes. The bubble size represents the number of associations found between intervention and outcome groups.

Figure 2 presents a partial visual representation of the EGM, with the distribution of associations between teledentistry interventions and health and education outcomes. The following outcomes were highlighted: ICT, e-learning, and tele-education.

The map represents the evidence that analyzed the effect of 43 different interventions in teledentistry organized into 8 groups: combined interventions, e-learning and tele-education, teleconsultation and teleservice, telemonitoring, telediagnosis, telescreening, ICTs, and AI—for at least 1 of the 71 health and education outcomes. Furthermore, these outcomes were organized into 8 different groups: diagnosis accuracy, education and professional training, user behavior, clinical practice, patient-centered outcomes, clinical outcomes, health services management, and access to health services. The interactive map—with confidence level, effects, findings, and development information—can be assessed in the study by Portella et al [17].

**Table 4:** Reviews with a high level of confidence included in the map along with focus populations and main positive and potentially positive effects.

Reference number of the included study, quoted in Multimedia Appendix 2	Focus population	Main positive and potentially positive associations
16	Parents	mHealth^a^ was found to have a potentially positive effect on parents’ health education
43	Undergraduate students	The combined action of tele-education and virtual reality was found to be potentially positive to the learning process of undergraduate students and led to cost reduction within health services management
48	Dental surgeons and patients receiving orthodontic treatment	Technology-based interventions were found to be positive for patient monitoring and different user behavior–related outcomes (adherence to ICT^b^, ICT satisfaction, and tool use) and potentially positive for the management of health emergencies and a number of clinical practice outcomes (when applied to oral surgery, communication among professionals, and professional-patient communication) as well as ICT reliability.
60	Patients with cancer	Telemonitoring via application was proven to improve service quality and access to health services among patients with cancer and to increase the levels of adherence to ICT, ICT satisfaction, and tool use in user-related outcomes.
61	Patients (orthodontic)	mHealth was found to be positive for the expansion of health care and adherence to orthodontic treatment. Potentially positive effects were seen when mHealth was applied to the improvement of clinical outcomes in general, health behavior change among patients, and when applied to oral surgery.
62	Dental students	Tele-education on an emergency basis was proven to positively improve learning and both perception and satisfaction with ICT. It has also shown the potential of improving pedagogical models and the tool use experience.
8	Patients (adults and children)	Multiple approaches in teledentistry have shown to be potentially positive on the regulation and management of health care systems. Telediagnosis through clinical photography and digital cameras was found to have a potentially positive effect on the diagnosis of potentially malignant oral lesions, allied to the potential of telemonitoring on improving patients’ prognosis.
11	Parents and guardians	Technology-based interventions were found to be effective in the reduction of gingival inflammation and plaque index levels and the improvement of patients’ health education.
17	Patients (children, adolescents, adults, and older individuals)	Either through a smartphone or clinical photography, telediagnosis was shown to have a positive effect on children, adolescents, adults, and older individuals.
19	Patients (children and adults)	The use of artificial intelligence in the diagnosis of carious lesions was found to be positive through both professional camera pictures and intraoral cameras. A potentially positive effect was found for the same application via smartphone pictures.
23	Dental students and dentists	The use of virtual reality reflected positive outcomes for the improvement of clinical skills and educational paths as well as for applications on restorative dentistry and ICT satisfaction levels.
31	Patients (children and adolescents)	e-Learning interventions were found to have a positive effect on the reduction of plaque index and probing depth as well as on patients’ health behavior change and education.
33	Patients (children)	The use of digital distraction through virtual reality had a positive effect on children in terms of treatment behavior, anxiety relief, and pain perception reduction.
36	Dental students	Digital approaches in dental education were found to be positive for the following outcomes: learning, assessment performance, professional training, students’ stress management, and students’ convenience. The level of ICT satisfaction was also positive.
39	Patients receiving orthodontic treatment (children and adolescents)	The results have shown positive effects for eHealth technologies and telemonitoring through apps directed at the improvement of clinical outcomes in general, decreasing the number of appointments and procedure planning in orthodontic treatments. Telemonitoring, in general, has proven to be potentially positive for these same outcomes.
44	Patients	The use of telecare, technology-based interventions, and social media was found to improve oral disease prevention, promote health behavior change, and increase treatment adherence among patients.
55	Patients (adults)	The combination of telescreening with telediagnosis, the use of multiple approaches in teledentistry, telediagnosis (through smartphone or clinical photography), and the use of social media have shown positive effects on the diagnosis of cancerous and potentially malignant oral lesions, on the prevention of oral diseases, in prognosis improvement, and in decision-making in clinical practice.
52	Patients receiving orthodontic treatment	Telemonitoring in general was found to have a positive effect when applied to oral surgery and on treatment adherence. Technology-based interventions have shown positive potential on changing patients’ hygiene habits and potentially positive on treatment adherence, while the use of social media was found to have a positive effect on professional-patient communication and potentially positive on treatment adherence.
37	Patients (adults) and dental students	Digital approaches in dental education, hybrid teaching within tele-education, and technology-based interventions were proven to enhance learning, improve professional training, and increase clinical skills and students’ confidence.

^a^mHealth: mobile health.

^b^ICT: information and communication technology.

Among the groups of outcomes, the education and professional training group stands out (97/494, 19.6%), followed by the health services management group (60/494, 12.1%) and patient-centered outcomes (74/494, 15%). The following outcomes were highlighted: learning (26/494, 5.3%), clinical skills improvement (24/494, 4.8%), health care extension (21/494, 4.2%), professional training (21/494, 4.2%), oral disease prevention (19/494, 3.8%), time optimization (18/494, 3.6%), and health education (16/494, 3.2%).

As for the 494 associations, the ICTs group stands out with 182 (37%) associations. Of the 494 associations, interventions with e-learning or tele-education, telediagnosis, and combined interventions appear with 96 (19.4%), 67 (13.5%), and 53 (10.7%) associations, respectively. A smaller number of associations were observed for telemonitoring (44/494, 8.9%), teleconsultation and teleservice (36/494, 7.3%), AI (9/494, 1.8%), and telescreening (7/494, 1.4%) interventions.

Regarding the publication countries (in which the publication journal is based), most of the SRs were from the United States (22/68, 32%), Switzerland (12/68, 18%), United Kingdom (10/68, 15%), England (9/68, 13%), along with 13% (9/68) from other countries. The focus countries (the countries and populations where the primary studies included in the SRs were conducted) retrieved from the SRs were mostly concentrated in Europe (42/68, 62%), Asia (40/68, 59%), and North America (36/68, 53%). In total, 28% (19/68) of the SRs did not specify the focus country.

As for the study populations, of the 68 studies, most studies had their interventions aimed at patients: 19 (28%) SRs focused on children and pediatric patients, 17 (25%) on adults, 13 (34%) on patients or “general population,” 7 (10%) on adolescents, and 3 (4%) on older adults. Other studies specified the group of patients based on their clinical condition: 1% (1/64) of the studies included patients with cancer, 3% (2/68) of the studies included patients who have undergone surgery, and 10% (7/68) of the studies included patients receiving orthodontic treatment. Of the 68 studies, dental students were the chosen population in 19 (28%) studies; dental surgeons or dentists in 11 (16%) studies; health professionals and provisioners in 5 (7%) studies; mothers, parents, relatives, or caregivers in 5 (7%) studies; and teachers and academicians in 1 (1%) study.

The most prevalent study designs included in the SRs were observational studies (32/68, 47%), including cohort, case series, cross-sectional, and case-control studies, and randomized controlled trials (30/68, 44%), followed by non-randomized controlled trials (10/68, 15%), experimental or quasi-experimental studies (8/68, 12%), and qualitative studies (5/68, 7%).

### Interventions for Outcome Groups

#### Overview

Among the groups of outcomes, out of 494 associations, the education and professional training group stands out with 97 (20%) associations, followed by the clinical practice group with 75 (15%) associations, and patient-centered outcomes group with 74 (15%) associations. Among the specific outcomes, highlights include learning and clinical skills improvement included in the educational and professional training group, with 26 (5.3%) and 26 (5.3%) associations, respectively; health care extension included in the health services management group, with 21 (4.3%) associations; oral disease prevention included in the clinical outcomes group, with 19 (3.8%) associations; and health education, included in the patient-centered outcomes group, with 16 (%) associations.

**Figure 3:**
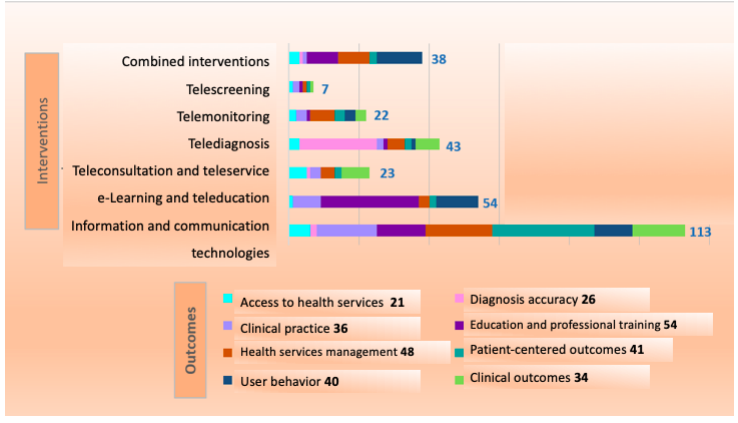
Distribution of outcomes among the 8 teledentistry intervention groups.

As for the interventions, the ICT group stands out with 182 (37%) associations from the 494, with emphasis on the following specific interventions: technology-based interventions (n=54,10.9% associations), mobile health (n=32, 6.5% associations), and digital distraction and virtual reality (n=24, 4.9% associations). The e-learning and tele-education group comes second with 96 (19%) associations out of 494 associations. Telediagnosis in general, telemonitoring in general, and digital approaches in dental education are the other highlights among the specific interventions, with 30 (6%) associations for the first and 27 (5.5%) associations for the 2 others. Figure 3 presents the distribution of outcomes for the teledentistry intervention groups included in the map.

#### Group 1: Access to Health Services

The 6 outcomes of the access to health services group received 21 (4.3%) associations of the total 494 associations. The most common outcome in the group was *service quality* (with 10/494, 2% associations), and no intervention prevalence was found, indicating a great variety of interventions aimed at the improvement of service provision (eg, teleconsultation, telescreening, telediagnosis, and social media). Of these 10 associations for service quality, 8 (80%) reported a positive or potentially positive effect (confidence level: 7 critically low and 1 high) and 2 (20%) did not present reported effects.

#### Group 2: Clinical Outcomes

The 9 specific outcomes of the clinical outcomes group received 66 (13.4%) associations of the total 494 associations. The following outcomes were highlighted: oral disease prevention (19/494, 3.8% associations), prognosis improvement (13/494, 2.6% associations), plaque index reduction (11/494, 2.2% associations), and gingival inflammation reduction (10/494, 2% associations). These prevalent outcomes sum up 53 associations of which 51 (96%) present positive or potentially positive effects (1 inconclusive and 1 no effect) and 18 (34%) belong to high confidence–level reviews based on the AMSTAR2 criteria. The most common intervention group associated with clinical outcomes was ICT, with 27 (5.5%) associations out of 494—22 (81%) of those presenting positive or potentially positive effects and 9 (27%) of those belonging to high confidence–level reviews.

#### Group 3: Clinical Practice

The 12 specific outcomes of the clinical practice group received 75 (15.2%) associations of the total 494 associations. The most common outcome was applied to orthodontics (14/494, 2.8% associations), followed by decision-making (13/494, 2.6% associations), clinical training (9/494, 1.8% associations), and digital planning (8/494, 1.6% associations). These 4 outcomes are responsible for 44 associations of multiple confidence level reviews (18 critically low, 13 low, 8 high, and 5 moderate). The main intervention was ICT, with 27 associations, of which 23 (85%) reported a positive or potentially positive effect (confidence level: 9 critically low, 8 high, 5 low, and 1 moderate).

#### Group 4: Diagnosis Accuracy

The 10 outcomes of the diagnosis accuracy group received 44 (8.9%) associations of the total 494 associations. The following outcomes were highlighted: carious lesions (10/494, 2% associations), diagnosis in general (8/494, 1.6% associations), cancer (7/494, 1.2% associations), and potentially malignant lesions (7/494, 1.2% associations). The main intervention was, naturally, telediagnosis, with 30 (6%) associations out of 494, of which 26 (87%) reported a positive or potentially positive effect (confidence level: 18 critically low and 8 high), 2 (7%) were inconclusive (both critically low), and 2 (7%) associations presented a negative report (both critically low).

#### Group 5: Education and Professional Training

The 9 outcomes delivered by the education and professional training group received 97 (19.6%) associations of the total 494 associations. A prevalence of the following specific outcomes was perceived: learning (26/494, 5.3% associations), clinical skills improvement (24/494, 4.9% associations), and professional training (21/494, 4.3% associations). Although the most common intervention was e-learning and tele-education, with 58 (11.7%) out of 494 associations, a substantial number of associations occurred for the ICT group (24/494, 4.9% associations). By combining these 2, a total of 74 (90%) of the 82 associations reported a positive or potentially positive effect (confidence level: 31 critically low, 21 high, 21 low, and 1 moderate), 7 (9%) were found to be inconclusive (confidence level: 5 critically low, 1 low, and 1 moderate), and 1 (1%) did not present any effects (confidence level: critically low).

#### Group 6: Health Services Management

The 7 outcomes of the health services management group received 60 (12%) associations of the total 494 associations. The following outcomes were highlighted: health care extension (21/494, 4.3% associations), time optimization (18/494, 3.6% associations), and cost reduction (7/494, 1.4% associations). The main intervention groups were ICT—with 21 (4.3%) associations—and telemonitoring—with 12 (2.4%) associations. Among these 33 associations, 26 (79%) have presented a positive or potentially positive effect (confidence level: 18 critically low, 6 high, and 2 moderate), 3 (9%) have proven to have no effect (confidence level: 2 moderate and 1 high), 2 (6%) have no reported effect (both critically low confidence), and 2 (6%) were found to be inconclusive (both low confidence).

#### Group 7: Patient-Centered Outcomes

The 9 outcomes of the patient-centered outcomes group received 74 (15%) associations of the total 494 associations. A combination of different specific outcomes was found; of the 74 associations, there were 16 (22%) associations linked to health education, 14 (19%) to health behavior change, 13 (18%) aimed at anxiety reduction, 12 (16%) at pain perception reduction, and 9 (12%) at hygiene habits change. The outstanding intervention group linked to this group was ICT, with 50 (10.1%) out of the 494 associations. In total, 74% (37/50) of these have positive or potentially positive effects (confidence level: 18 critically low, 11 high, 4 moderate, and 1 low), 5 (10%) were inconclusive (confidence level: 3 high and 1 critically low), 5 (10%) have no reported effect (confidence level: 4 critically low and 1 high), and 3 (6%) did not show any effect (confidence level: 2 high and 1 low).

#### Group 8: User Behavior

The 8 outcomes of the user behavior group received 57 (12%) associations of the total 494 associations. A prevalence of the following specific outcomes within the user behavior group was found: ICT satisfaction (11/57, 19% associations), tool use (11/57, 19% associations), treatment adherence (10/57, 18% associations), and ICT acceptability (9/57, 16% associations). Associations linked to the intervention groups were sparse and mostly distributed between ICT (23/57, 40% associations), e-learning and tele-education (13/57, 23% associations), and combined interventions (13/57, 23% associations). These 3 groups resulted in 49 (9.9%) associations out of 494, of which 44 (89.8%) were positive or potentially positive (confidence level: 24 critically low, 15 high, 4 low, and 1 moderate), 3 (6%) have shown no effect (confidence level: all high), 1 (2%) did not report an effect (confidence level: critically low), and 1 (2%) has presented a potentially negative effect (confidence level: high).

### Interventions With Teledentistry

The teledentistry evidence map brings together studies of interventions with 4 combined interventions, 9 e-learning and tele-education actions, 11 ICT approaches aimed at teledentistry, 4 teleconsultation and teleservice actions, 4 telediagnosis, 5 telemonitoring, 2 telescreening, and 2 AI distinct approaches. The effect of these teledentistry interventions was analyzed on 494 associations for 71 health and education outcomes, and the group with the highest number of associations was the ICT, with 182 associations (37%).

The most researched approach on teledentistry reported by the map was technology-based interventions, with 54 unit interventions aimed at a great range of outcomes but mostly at user behavior, with 12 (22%) associations (11 of those reporting positive or potentially positive effects); health services management, with 10 (19%) associations (9 of which presenting positive or potentially positive effects); education and professional training, with 8 (15%) associations (all 8 of those reporting positive effect); and clinical practice, with 8 (15%) associations (all 8 reporting positive or potentially positive effects). The second most researched approach on teledentistry was mobile health, with 32 unit interventions also directed at a considerable variety of outcomes but more substantially focused on clinical outcomes, with 14 (44%) associations (10 reporting positive or potentially positive effects), and patient-centered outcomes, with 9 (28%) associations (5 of those reporting positive or potentially positive effects, 1 with no given effect, 1 not reported, and 2 inconclusive).

Most of the studies (67/68, 99%) have presented at least 1 positive or potentially positive association with teledentistry interventions. The only exception refers to the inconclusive results of a meta-analysis focused on digital technology distraction for acute pain in children, to which the given AMSTAR2 confidence level was critically low. Among the studies that have achieved a high level of confidence on AMSTAR2, all have reported at least 1 positive or potentially positive association with regard to teledentistry interventions.

## Discussion

### Principal Findings

This EGM is based on 68 (SRs: n=52, 77%; SRs with meta-analysis: n=15, 22%; and metanalysis: n=1, 2%) studies that met the established inclusion criteria, and it provides a broad overview of available evidence on teledentistry interventions, applied to 64 outcomes in health and education. The majority of focus countries identified in the SRs are located in Europe (42/68, 62%), Asia (40/68, 59%), and North America (36/68, 53%). This suggests a need to study regions where online dental services could be beneficial, to address potential social and economic limitations in those areas.

While a great variety of study designs was found among the primary studies presented in the included reviews, there was a substantial prevalence of randomized controlled trials (31/68, 46%), followed by a range of observational studies (20/68, 29%). This finding endorses a higher level of design reliability, ultimately giving more scientific strength to the EGM itself. The map shows the volume of available research and highlights areas where the interventions have presented positive or potentially positive effects, signaling a broad range of not only research but also service implementation possibilities [89].

By restricting the results obtained by “high confidence level” reviews (based on the AMSTAR2 criteria) and graphically represented in the map, the incorporation of teledentistry into the teaching-service axis proposed by Chen et al [10] becomes a strong evidence-based material, especially because these high confidence–level reviews pointed to 121 (89%) out of 136 positive or potentially positive associations for teledentistry interventions [89]. The use of the AMSTAR2 tool refines the selection of articles through its specific and rigorous methodology, which focuses on the structural aspects of study designs and protocols. While this may exclude some articles with relatively good methodology and results, using AMSTAR2 is still crucial as it strengthens the overall EGM. Therefore, the EGM methodology enables the transformation of the qualified evidence found into practice in a markable accessible format, creating an important conceptual connection with evidence-based best practices. Its interface tends to be more visually compact and dynamic when compared to other systematic study designs, especially for professionals outside the academic and research realm, such as policy makers and service managers, thereby enhancing outcomes and creating cross-references that serve as a reliable index to understanding and planning optimized service offering to the population.

For students, teledentistry applications enable the improvement of the learning process, convenience, clinical skills, and professional training as well as increase the levels of perception, confidence, and satisfaction with ICT. Furthermore, teledentistry enhances assessment performance and improves pedagogical models and dental curricula. At the professional level, it helps dentists refine and optimize their clinical practice, manage emergencies, and plan procedures. Evidence indicates distinct benefits in orthodontics, restorative dentistry, and oral surgery practices. Better time management and team organization (generating less burden) are also among the operational benefits for dental services [21-25].

For patients and caregivers, teledentistry improves health education and promotion as well as the quality of service provision (from cost reduction to professional-patient communication). It can increase the levels of adherence to dental treatments and clinical outcomes in general, endorsing health behavior change, managing anxiety and pain perception, and favoring better prognosis for all types of patients [26-38]. Within health services, teledentistry promotes reductions in cost and number of appointments, improves time and emergency management, and facilitates the access to specialists [22,35,39].

Updating and improving requirements and compliances become essential for the consistent and broad development of teledentistry and for improving oral health provision [91]. As for the education and training of dental students, Kröplin et al [92] endorsed the need for digital health topics to be taught at universities in a transdisciplinary manner, developing the curricula and enabling the proper use of digital health platforms by creating an adequate scenario for the progressive use of ICTs in general.

Online learning methodology attached to a hybrid approach to care is an attractive, flexible, and convenient option for learners, with lower costs, larger geographic coverage, and easy access to information. These gains apply for undergraduate students, graduate students, and dentists in continuous medical education programs, endorsing tele-education as an alternative to keeping dental professionals up to date with the latest advancements in their field and, thus, benefiting the patients they serve [92].

The data collected demonstrate that this experience is a safe practice tool that enhances both educational and professional development. It aligns with the growing digital use and literacy in society as well as the trend in health services toward hybrid care (synchronized in-person and online offerings). This approach provides a more accessible and inclusive pathway to evidence-based practices [16].

Another important aspect to be considered is the regulatory mainframe. The speed of social, cultural, and health changes in society is usually faster than the effort of legislators and policy makers to address new forms of offering health and dental services, which is understandable, due to the time needed for a coherent decision path, based on collecting reliable data, analysis, broad discussion, and finally document writing and approval. However, security concerns and the demand for guidelines and protocols must be taken into account, as stated by Hung et al [91]. Those points can lead to some friction in the patient-provider relationship. Therefore, private offices and public services need to be aware of the impacts and consequences of offering virtual service solutions, following consistent operational protocols, and using safe virtual environments. Thus, to provide and extend dental care through teledentistry, clinician and patient training must rely on national laws and regulations; challenges such as the inability to perform diagnostic tests and the potential for misdiagnosis due to variations in image and video quality must be carefully considered [91].

In addition, it is important to consider the complex landscape across each continent, where various countries—and even their provinces and states—interact with one another. This creates a paradox of having a wide range of laws while also addressing specific local needs, along with somewhat independent health care systems. This is observed, for instance, in Europe (in the European Union and each member country) as well as in the United States (that has a good level of legal independence among its states). In this context, it is observed that the major updated national laws (or regulations) in several parts of the world address individual and health data management but in a more generalized approach, such as the Health Insurance Portability and Accountability Act (United States), the General Data Protection Regulation Act (European Union), the Data Protection General Law (Brazil), and the Act on the Protection of Personal Information (Japan), among others. Specific segments of the health care system (including dentistry) have to create their own guidelines in an effort to align with those bigger legislations [91,93-102]. One of Pan American Health Organization’s primary goals in developing and publishing the EGM is to provide consistent and clear information for policy makers and public service managers. This includes the ability to filter results by different countries and populations, with the aim of improving dental services for citizens and communities, ultimately creating tangible benefits for society as a whole [103].

From the patient’s perspective, it is a simpler train of thought, focused on the expectation that a reliable, swift, and effective teledentistry treatment could be offered. There is also a perception that health care providers and governments already observed and considered all legal and safety aspects. In summary, the high-confidence SRs included in this EGM (cited in Table 4 and referenced in Multimedia Appendix 2), demonstrate the benefits of teledentistry interventions in favor of the fulfillment of needs perceived by several different populations and focus countries. Positive (or potentially positive) associations regarding the focus populations (parents, patients, and dental students) and outcomes, such as greater adherence to oral cancer treatments, gingival inflammation reduction, better orthodontic treatment outcome, diagnosis of oral lesions, and learning and skills improvement among dental students are presented in Table 4. These examples demonstrate the real impact of teledentistry through evidence-based data, especially toward clinical and oral health benefits in individuals (patients), serving as an important reference to policy makers and dental service managers.

### Limitations

The results of this study are aligned with those of Yan et al [34], Uhrin et al [37], Erdilek et al [21], Dedeilia et al [24], and Bandiaky et al [25] regarding the effectiveness of teledentistry interventions toward health and education outcomes. As the methodology does not exclude duplicates among the primary studies individually included in the SRs, it is important to acknowledge the risk of amplification of evidence reported for a specific association (with a consequent increase in the bubble size represented in the interactive map, working as a nonintentional reinforcement visualization). Moreover, although a growth in the AMSTAR2 confidence level is perceived in most recently published SRs, the prevalence of “critically low” quality studies included in this EGM has to be considered to guide the development of actions and policies as well as the criteria of new teledentistry-themed reviews. Protocols attending to data privacy and global legal support are also essential to strengthen the great range of teledentistry applications and to acknowledge the importance of maintaining face-to-face health provision [95-98,101,104].

The reviews analyzed in this study either did not explore regulation or did not consider it as a core aspect of analysis. Therefore, more studies should be conducted in the future, specifically to understand those interferences and outcomes. Furthermore, although SRs provide a gold-standard panorama for a certain subject, other study designs and recent primary studies can reflect different experiences with teledentistry that have not been taken into consideration for the map development.

### Conclusions

The results and graphic representations found in this EGM serve as a user-friendly instrument and a dynamic interface for the comprehension of the applications of teledentistry toward health and education outcomes, thus illustrating the answers to the proposed research question. Navigating through the interactive map allows users to understand where the reliable associations can be found or where a lack of high-quality evidence is perceived, directing the search to specific interventions, outcomes, focus populations, focus countries, etc, as needed.

The publication and further analysis of the teledentistry EGM results provide an accessible pathway to evidence-based policy making, pedagogical enhancement, and patient care. The great variability found between interventions and health and educational outcomes is noticeable and could endorse the inclusion of ICT in modern dentistry and teaching. This scenario underscores the potential of teledentistry as a comprehensive tool for improving the teaching-learning process and enhancing clinical skills within dental curricula as well as for qualifying health service provision.

## Appendix

Multimedia Appendix 1 Search strategy.

Multimedia Appendix 2 References for the included studies.

Multimedia Appendix 3 Studies excluded after full-text reading and reasons for exclusion.

## Abbreviations

AI: artificial intelligence
AMSTAR2: A Measurement Tool to Assess Systematic Reviews
BIREME/OPAS: Latin American and Caribbean Center for Health Sciences Information of the Pan American Health Organization
EGM: evidence gap map
ICT: information and communication technology
PRISMA: Preferred Reporting Items for Systematic Reviews and Meta-Analyses
SR: systematic review
