# Correction: Teledentistry Applied to Health and Education Outcomes: Evidence Gap Map

**DOI:** 10.2196/71577

**Published:** 2025-02-03

**Authors:** Júlia Nascimento da Silva Mulder, Marcelo Ramos Pinto, Isabelle Aníbal, Ana Paula Dornellas, Deise Garrido, Camila Huanca, Ana Estela Haddad, Carmen Verônica Mendes Abdala

**Affiliations:** 1 Department of Orthodontics and Pediatric Dentistry University of São Paulo - School of Dentistry São Paulo Brazil; 2 Latin American and Caribbean Center on Health Sciences Information São Paulo Brazil

In “Teledentistry Applied to Health and Education Outcomes: Evidence Gap Map” (JMIR 2024;26:e60590) the authors made one addition.

In the Acknowledgements paragraph, the text in **bold** has been added:

This study was carried out with financial support from the National Council for Scientific and Technological Development (Cnpq) – Federal funds from the Brazilian government granted by the Process: 422896/2021-7; CNPq/MCTI/FNDCT Call No. 18/2021—Track A—Emerging Groups, **as well as from the CNPq productivity grant project bench fund CNPq Call No. 03/2021 - Technological Development and Innovative Extension (DT) Grants, Process: 305972/2021-9.** The main author (JNdSM) also acknowledges the financial support from the Coordination of Superior Level Staff Improvement, Brazil. This map applies the evidence gap map methodology supported by the Latin American and Caribbean Center on Health Sciences (BIREME/OPAS/OMS), in partnership with the Telehealth Center FOUSP-SAITE (School of Dentistry, University of São Paulo).

The correction will appear in the online version of the paper on the JMIR Publications website on February 3, 2025, together with the publication of this correction notice. Because this was made after submission to PubMed, PubMed Central, and other full-text repositories, the corrected article has also been resubmitted to those repositories.

