# Supplementary material for: Teledentistry Applied to Health and Education Outcomes: Evidence Gap Map
Source: J Med Internet Res. 2024 Nov 27;26:e60590. doi: 10.2196/60590 (PMC11635335; doi:10.2196/60590)
Supplement: Multimedia Appendix 1 [file jmir_v26i1e60590_app1.docx]

**APPENDIX 1 - SEARCH STRATEGY**

| **PUBMED - https://pubmed.ncbi.nlm.nih.gov/** | | |
| --- | --- | --- |
| #1 | Telehealth | ("communication technologies" OR "Consulta a distancia" OR "consulta remota" OR "Data security" OR "Data storage" OR "digital health" OR "digital clinical examinations" OR "distance deliver care" OR "e-assessments" OR "educational electronic platforms" OR "e-health" OR "e-learning" OR "Electronic medical prescription" OR "Electronic monitoring" OR "e-salud" OR "e-saúde" OR ehealth OR esalud OR esaúde OR "e-teaching" OR "Health Teleservices" OR "Health Tele-services" OR "Health, Mobile" OR "m-health" OR mhealth OR "mobile application" OR "mobile applications" OR "Mobile Health" OR "monitoring of patients" OR "Remote advice" OR "remote assessment" OR "Remote Consultation" OR "Remote consultations" OR "remote continuity of care" OR "Remote counseling" OR "remote examination" OR "Remote guidance" OR "Remote prescriptions" OR "Remote referrals" OR "remote screening" OR "remote triaging" OR "saúde digital" OR "salud digital" OR "tele assessment" OR "tele assistance" OR teleassistencia OR Teleconsulta OR teleconsultation OR Teleconsultoria OR Teleconsultorias OR telecuidado OR telecure OR Telecare OR Telediagnosis OR Telediagnóstico OR "tele educação" OR teleducação OR telehealth OR telemedicina OR telemedicine OR Telemonitoramento OR Telemonitoring OR Telemonitorización OR Teleorientação OR Teleorientación OR Teleorientation OR Telepreceptoria OR teleradiology OR telesalud OR teleservice* OR telessaúde OR "telecommunication technologies" OR "Virtual appointment" OR "virtual consultations" OR "virtual disease management" OR "Virtual meetings" OR "Virtual tools" OR "virtual triage" OR "Virtual vigilance" OR "Virtual visits") |
| #2 | Dentistry | ("academic dentistry" OR "clinical dentistry" OR new normal clinical dentistry OR "continuous dental care" OR "dental care" OR "dental education" OR "educação odontológica" OR "oral care" OR "oral health care" OR "oral health surveillance" OR "oral health" OR "oral services" OR "saúde bucal" OR "saúde oral" OR Dentistry OR prosthodontics) |
| #3 | Teledentistry | ("remote dental care" OR "remote dental screening" OR "remote dental telehealth" OR "remote dentistry" OR Teleodontologia OR teledentistry OR "e-dentistry" OR "teledentistry policies" OR "virtual dental care" OR "virtual dentistry" OR "social distancing dentistry") |
| #4 | (Telehealth AND Dentistry) OR Teledentistry | (#1 AND #2) OR #3 |
|  | Filters | Systematic Reviews + Metanalysis |

|  |  |  |
| --- | --- | --- |
| **Virtual Health Library - http://bvsalud.org** | | |
| **#1** | **Telehealth** | ("communication technologies" OR "Consulta a distancia" OR "consulta remota" OR "Data security" OR "Data storage" OR "digital clinical examinations" OR "digital health" OR "distance deliver care" OR "e-assessments" OR "educational electronic platforms" OR "e-health" OR "e-learning" OR "Electronic medical prescription" OR "Electronic monitoring" OR "e-salud" OR "e-saúde" OR ehealth OR esalud OR esaúde OR "e-teaching" OR "Health Teleservices" OR "Health Tele-services" OR "Health, Mobile" OR "m-health" OR mhealth OR "mobile application" OR "mobile applications" OR "Mobile Health" OR "monitoring of patients" OR "Remote advice" OR "remote assessment" OR "Remote Consultation" OR "Remote consultations" OR "remote continuity of care" OR "Remote counseling" OR "remote examination" OR "Remote guidance" OR "Remote prescriptions" OR "Remote referrals" OR "remote screening" OR "remote triaging" OR "saúde digital" OR "tele assessment" OR "tele assistance" OR teleassistencia OR Teleconsulta OR teleconsultation OR Teleconsultoria OR Teleconsultorias OR telecuidado OR telecure OR Telecare OR Telediagnosis OR Telediagnóstico OR "tele educação" OR teleducação OR telehealth OR telemedicina OR telemedicine OR Telemonitoramento OR Telemonitoring OR Telemonitorización OR Teleorientação OR Teleorientación OR Teleorientation OR Telepreceptoria OR teleradiology OR telesalud OR teleservice* OR telessaúde OR "telecommunication technologies" OR "Virtual appointment" OR "virtual consultations" OR "virtual disease management" OR "Virtual meetings" OR "Virtual tools" OR "virtual triage" OR "Virtual vigilance" OR "Virtual visits") |
| **#2** | **Dentistry** | ("academic dentistry" OR "clinical dentistry" OR new normal clinical dentistry OR "continuous dental care" OR "dental care" OR "dental education" OR "educação odontológica" OR "oral care" OR "oral health care" OR "oral health surveillance" OR "oral health" OR "oral services" OR "saúde bucal" OR "saúde oral" OR Dentistry OR prosthodontics) |
| **#3** | **Teledentistry** | ("remote dental care" OR "remote dental screening" OR "remote dental telehealth" OR "remote dentistry" OR Teleodontologia OR teledentistry OR "e-dentistry" OR "teledentistry policies" OR "virtual dental care" OR "virtual dentistry" OR "social distancing dentistry") |
| **#4** | **(Telehealth AND Dentistry) OR Teledentistry** | **(#1 AND #2) OR #3** |
|  | **Exclusion** | **DB:"MEDLINE"** |
|  | **Filters** | **Systematic Reviews + Metanalysis** |

|  |  |  |
| --- | --- | --- |
| **EMBASE - https://www.periodicos.capes.gov.br** | | |
| **#1** | **Telehealth** | communication technologies':ab,ti OR 'data security':ab,ti OR 'data storage':ab,ti OR 'digital health':ab,ti OR 'digital clinical examinations':ab,ti OR 'distance deliver care':ab,ti OR 'e-assessments':ab,ti OR 'educational electronic platforms':ab,ti OR 'e-health':ab,ti OR 'e-learning':ab,ti OR 'electronic medical prescription':ab,ti OR 'electronic monitoring':ab,ti OR 'e-salud':ab,ti OR 'e-saúde':ab,ti OR ehealth:ab,ti OR esalud:ab,ti OR esaúde:ab,ti OR 'e-teaching':ab,ti OR 'health teleservices':ab,ti OR 'health tele-services':ab,ti OR 'health, mobile':ab,ti OR 'm-health':ab,ti OR mhealth:ab,ti OR 'mobile application':ab,ti OR 'mobile applications':ab,ti OR 'mobile health':ab,ti OR 'monitoring of patients':ab,ti OR 'remote advice':ab,ti OR 'remote assessment':ab,ti OR 'remote consultation':ab,ti OR 'remote consultations':ab,ti OR 'remote continuity of care':ab,ti OR 'remote counseling':ab,ti OR 'remote examination':ab,ti OR 'remote guidance':ab,ti OR 'remote prescriptions':ab,ti OR 'remote referrals':ab,ti OR 'remote screening':ab,ti OR 'remote triaging':ab,ti OR telecare:ab,ti OR telediagnosis:ab,ti OR telehealth:ab,ti OR telemonitoring:ab,ti OR telemonitorización:ab,ti OR teleorientation:ab,ti OR telepreceptoria:ab,ti OR teleradiology:ab,ti OR telesalud:ab,ti OR teleservice:ab,ti OR 'telecommunication technologies':ab,ti OR 'virtual appointment':ab,ti OR 'virtual consultations':ab,ti OR 'virtual disease management':ab,ti OR 'virtual meetings':ab,ti OR 'virtual tools':ab,ti OR 'virtual triage':ab,ti OR 'virtual vigillance':ab,ti OR 'virtual visits':ab,ti |
| **#2** | **Dentistry** | academic dentistry':ab,ti OR 'clinical dentistry':ab,ti OR 'new normal clinical dentistry':ab,ti OR 'continuous dental care':ab,ti OR 'dental care':ab,ti OR 'dental education':ab,ti OR 'oral care':ab,ti OR 'oral health care':ab,ti OR 'oral health surveillance':ab,ti OR 'oral health':ab,ti OR 'oral services':ab,ti OR dentistry:ab,ti OR prosthodontics:ab,ti |
| **#3** | **Teledentistry** | ('remote dental care':ab,ti OR 'remote dental screening':ab,ti OR 'remote dental telehealth':ab,ti OR 'remote dentistry':ab,ti OR teledentistry:ab,ti OR 'e-dentistry':ab,ti OR 'teledentistry policies':ab,ti OR 'virtual dental care':ab,ti OR 'virtual dentistry':ab,ti OR 'social distancing dentistry':ab,ti) |
| **#4** | **(Telehealth AND Dentistry) OR Teledentistry** | (#1 AND #2) OR #3 |
|  | **Exclusion** | **[pubmed-not-medline]/lim** |
|  | **Filter** | **review'/it** |
