# Supplementary material for: Teledentistry Applied to Health and Education Outcomes: Evidence Gap Map
Source: J Med Internet Res. 2024 Nov 27;26:e60590. doi: 10.2196/60590 (PMC11635335; doi:10.2196/60590)
Supplement: Multimedia Appendix 2 [file jmir_v26i1e60590_app2.docx]

**APPENDIX 2 - REFERENCES FOR INCLUDED STUDIES**

| **REFERENCE NUMBER AND STUDY TITLE** | **PUBLICATION COUNTRY** | **CONFIDENCE LEVEL** | **AUTHORS** | **YEAR** |
| --- | --- | --- | --- | --- |
| [1] A systematic review and network meta-analysis of virtual reality, audiovisuals and music interventions for reducing dental anxiety related to tooth extraction | United Kingdom | Low | Hao T and Pang J and Liu Q and Xin P | 2023 |
| [2] A systematic review of the research evidence for the benefits of teledentistry | United States | Critically low | Estai M and Kanagasingam Y and Tennant M and Bunt S | 2018 |
| [3] A systematic review of the use of mHealth in oral health education among older adults | Switzerland | Low | Chau RCW and Thu KM and Chaurasia A and Hsung RTC and Lam WY | 2023 |
| [4] A systematic review of the use of virtual reality or dental smartphone applications as interventions for management of paediatric dental anxiety | England | Critically low | Cunningham A and McPolin O and Fallis R and Coyle C and Best P and McKenna G | 2021 |
| [5] A Systematic Review of Virtual Reality Therapeutics for Acute Pain Management | United States | Critically low | Dreesmann NJ and Su H and Thompson HJ | 2022 |
| [6] A Systematic Review on the Validity of Teledentistry | United States | Critically low | Alabdullah JH and Daniel SJ | 2018 |
| [7] Accuracy of dental images for the diagnosis of dental caries and enamel defects in children and adolescents: A systematic review | England | Critically low | Inês Meurer M and Caffery LJ and Bradford NK and Smith AC | 2015 |
| [8] Accuracy of remote examination for detecting potentially malignant oral lesions: a systematic review and meta-Analysis | United States | High | Lima TMNR and Moura ABR and Bezerra PMM and Valença AMG and Vieira TI and Santiago BM and Cavalcanti YW and de Sousa AS | 2023 |
| [9] An systematic review of e-learning outcomes in undergraduate dental radiology curricula-levels of learning and implications for researchers and curriculum planners | England | Critically low | Botelho MG and Agrawal KR and Bornstein MM | 2019 |
| [10] Analysis of Deep Learning Techniques for Dental Informatics: A Systematic Literature Review | Switzerland | Critically low | AbuSalim S and Zakaria N and Islam MR and Kumar G and Mokhtar N and Abdulkadir SJ | 2022 |
| [11] App-based oral health promotion interventions on modifiable risk factors associated with early childhood caries: A systematic review | Switzerland | High | Ajay K and Azevedo LB and Haste A and Morris AJ and Giles E and Gopu BP and Subramanian MP and Zohoori FV | 2023 |
| [12] Application of virtual reality in dental implants: a systematic review | United Kingdom | Low | Monaghesh E and Negahdari R and Samad-Soltani T | 2023 |
| [13] Applications of teledentistry in dental practice: a systematic review | Colombia | Critically low | Fortich-Mesa, Natalia and Hoyos-Hoyos, Vivi | 2020 |
| [14] Are Technology-Based Interventions Effective in Reducing Dental Anxiety in Children and Adults? A Systematic Review | United States | Critically low | Gujjar KR and van Wijk A and Kumar R and de Jongh A | 2019 |
| [15] Awareness, Knowledge, Attitude, and Practice of Teledentistry among Dental Practitioners during COVID-19: A Systematic Review and Meta-Analysis | Switzerland | Critically low | Lin GSS and Koh SH and Ter KZ and Lim CW and Sultana S and Tan WW | 2022 |
| [16] Can mHealth promotion for parents help to improve their children's oral health? A systematic review | England | High | Wang K and Yu KF and Liu P and Lee GHM and Wong MCM | 2022 |
| [17] Comparative evaluation of dental caries score between teledentistry examination and clinical examination: a systematic review and meta-analysis | United States | High | Priyank H and Verma A and Zama Khan DU and Prakash Rai N and Kalburgi V and Singh S | 2023 |
| [18] Dental Care Access and the Elderly: What Is the Role of Teledentistry? A Systematic Review | Switzerland | Critically low | Aquilanti L and Santarelli A and Mascitti M and Procaccini M and Rappelli G | 2020 |
| [19] Detecting dental caries on oral photographs using artificial intelligence: a systematic review | United Kingdom | High | Moharrami M and Farmer J and Singhal S and Watson E and Glogauer M and Johnson AEW and Schwendicke F and Quinonez C | 2023 |
| [20] Diagnostic accuracy of teledentistry in the detection of dental caries: a systematic review | United States | Critically low | Estai M and Bunt S and Kanagasingam Y and Kruger E and Tennant M | 2016 |
| [21] Digital Technology Distraction for Acute Pain in Children: A Meta-analysis | United States | Critically low | Gates M and Hartling L and Shulhan-Kilroy J and MacGregor T and Guitard S and Wingert A and Featherstone R and Vandermeer B and Poonai N and Kircher J and Perry S and Graham TAD and Scott SD and Ali S | 2020 |
| [22] Digital Undergraduate Education in Dentistry: A Systematic Review | Switzerland | Critically low | Zitzmann NU and Matthisson L and Ohla H and Joda T | 2020 |
| [23] Digitalization era of dental education: a systematic review | Poland | High | Erdilek D and Gümüştaş B and Güray Efes B | 2023 |
| [24] Education Technology in Orthodontics and Paediatric Dentistry during the COVID-19 Pandemic: A Systematic Review | Switzerland | Critically low | Patano A and Cirulli N and Beretta M and Plantamura P and Inchingolo AD and Inchingolo AM and Bordea IR and Malcangi G and Marinelli G and Scarano A and Lorusso F and Inchingolo F and Dipalma G | 2021 |
| [25] Educational escape rooms for healthcare students: a systematic review. | Scotland | Low | Quek LH and Tan AJQ and Sim MJJ and Ignacio J and Harder N and Lamb A and Chua WL and Lau ST and Liaw SY | 2023 |
| [26] Effect of mHealth in improving oral hygiene: A systematic review with meta-analysis | United States | Critically low | Toniazzo MP and Nodari D and Muniz FWMG and Weidlich P | 2019 |
| [27] Effectiveness of dental monitoring system in orthodontics: a systematic review | United Kingdom | Moderate | Sangalli L and Alessandri-Bonetti A and Dalessandri D | 2023 |
| [28] Effectiveness of E-Learning in Oral Radiology Education: A Systematic Review | United States | Moderate | Santos GN and Leite AF and Figueiredo PT and Pimentel NM and Flores-Mir C and de Melo NS and Guerra EN and De Luca Canto G | 2016 |
| [29] Effectiveness of haptic feedback devices in preclinical training of dental students-a systematic review | United Kingdom | Low | Patil S and Bhandi S and Awan KH and Licari FW and Di Blasio M and Ronsivalle V and Cicciù M and Minervini G | 2023 |
| [30] Effectiveness of the Distance Learning Strategy Applied to Orthodontics Education: A Systematic Literature Review | United States | Critically low | Lima MS and Tonial FG and Basei E and Brew MC and Grossmann E and Haddad AE and Rivaldo EG and Vargas IA and Bavaresco CS | 2019 |
| [31] Effectiveness of various methods of educating children and adolescents for the maintenance of oral health: a systematic review of randomized controlled trials | United Kingdom | High | Atif M and Tewari N and Saji S and Srivastav S and Rahul M | 2023 |
| [32] Effectiveness of Virtual Reality and Interactive Simulators on Dental Education Outcomes: Systematic Review | Germany | Critically low | Moussa R and Alghazaly A and Althagafi N and Eshky R and Borzangy S | 2022 |
| [33] Effectiveness of virtual reality distraction interventions to reduce dental anxiety in paediatric patients: a systematic review and meta-analysis | Netherlands | High | Yan X and Yan Y and Cao M and Xie W and O'Connor S and Lee JJ and Ho MH | 2023 |
| [34] Effectiveness of Virtual Reality Glasses as a Distraction for Children During Dental Care | Brazil | Moderate | Custódio NB and Costa FDS and Cademartori MG and da Costa VPP and Goettems ML | 2020 |
| [35] Efficacy of mobile health care in patients undergoing fixed orthodontic treatment: A systematic review | England | Critically low | Choi EM and Park BY and Noh HJ | 2021 |
| [36] Health worker education during the COVID-19 pandemic: global disruption, responses and lessons for the future-a systematic review and meta-analysis | United Kingdom | High | Dedeilia A and Papapanou M and Papadopoulos AN and Karela NR and Androutsou A and Mitsopoulou D and Nikolakea M and Konstantinidis C and Papageorgakopoulou M and Sideris M and Johnson EO and Fitzpatrick S and Cometto G and Campbell J and Sotiropoulos MG | 2023 |
| [37] Impact of haptic simulators in preclinical dental education: a systematic review | United States | High | Bandiaky ON and Lopez S and Hamon L and Clouet R and Soueidan A and Le Guehennec L | 2023 |
| [38] Implementation of virtual OSCE in health professions education: a systematic review | United Kingdom | Low | Chan SCC and Choa G and Kelly J and Maru D and Rashid MA | 2023 |
| [39] Is teledentistry effective to monitor the evolution of orthodontic treatment? A systematic review and meta-analysis | Brazil | High | Torres DKB and Santos MCCD and Normando D | 2023 |
| [40] Managing dental phobia in children with the use of virtual reality: a systematic review of the current literature | Switzerland | Critically low | Rosa A and Pujia AM and Docimo R and Arcuri C | 2020 |
| [41] MOBILE APP AND SOCIAL MEDIA-BASED INTERVENTIONS MAY HAVE A POSITIVE INFLUENCE ON THE BEHAVIOR OF ORTHODONTIC PATIENTS | United States | Critically low | Montasser MA | 2022 |
| [42] Monitoring of awake bruxism by intelligent app | United Kingdom | Low | Velásquez Ron B and Mosquera Cisneros V and Pazmiño Troncoso P and Rodríguez Tates M and Alvares Lalvay E and Chauca Bajaña L and Ordoñez Balladares A | 2022 |
| [43] Online eLearning for undergraduates in health professions: A systematic review of the impact on knowledge, skills, attitudes and satisfaction | Scotland | High | George PP and Papachristou N and Belisario JM and Wang W and Wark PA and Cotic Z and Rasmussen K and Sluiter R and Riboli-Sasco E and Tudor Car L and Musulanov EM and Molina JA and Heng BH and Zhang Y and Wheeler EL and Al Shorbaji N and Majeed A and Car J | 2014 |
| [44] Online social networks for prevention and promotion of oral health: a systematic review | Canada | High | de Oliveira Júnior AJ and Oliveira JM and Bretz YP and Mialhe FL | 2023 |
| [45] Patient satisfaction with e-oral health care in rural and remote settings: a systematic review | Canada | Low | Emami, E. and Harnagea, H. and Shrivastava, R. and Ahmadi, M. and Giraudeau, N. | 2017 |
| [46] Pivoting Dental Practice Management during the COVID-19 Pandemic-A Systematic Review | Switzerland | Critically low | Mahdi SS and Ahmed Z and Allana R and Peretti A and Amenta F and Nadeem Bijle M and Seow LL and Daood U | 2020 |
| [47] Potential of Internet of Medical Things (IoMT) applications in building a smart healthcare system: A systematic review | Netherlands | Critically low | Dwivedi R and Mehrotra D and Chandra S | 2022 |
| [48] Relevance of teleorthodontic tools: a systematic review of the literature / Pertinence des outils de téléorthodontie : une revue systématique de la littérature | France | High | Rouanet F and Masucci C and Khorn B and Oueiss A and Dridi SM and Charavet C | 2022 |
| [49] Role of Digital Media in Promoting Oral Health: A Systematic Review | United States | Critically low | Sharma S and Mohanty V and Balappanavar AY and Chahar P and Rijhwani K | 2022 |
| [50] Surgical training 20: A systematic approach reviewing the literature focusing on oral maxillofacial surgery - Part I | France | Critically low | Grall P and Ferri J and Nicot R | 2021 |
| [51] Surgical Training 20: A systematic approach reviewing the literature focusing on oral maxillofacial surgery - Part II | France | Critically low | Grall P and Ferri J and Nicot R | 2021 |
| [52] Tele-orthodontics and sensor-based technologies: a systematic review of interventions that monitor and improve compliance of orthodontic patients | United States | High | Wafaie K and Rizk MZ and Basyouni ME and Daniel B and Mohammed H | 2023 |
| [53] Teledentistry and mHealth for Promotion and Prevention of Oral Health: A Systematic Review and Meta-analysis | United States | Low | Fernández CE and Maturana CA and Coloma SI and Carrasco-Labra A and Giacaman RA | 2021 |
| [54] Teledentistry in the diagnosis of oral lesions: A systematic review of the literature | United States | Critically low | Flores APDC and Lazaro SA and Molina-Bastos CG and Guattini VLO and Umpierre RN and Gonçalves MR and Carrard VC | 2020 |
| [55] Teledentistry: a future solution in the diagnosis of oral lesions: diagnostic meta-analysis and systematic review | United States | High | Uhrin E and Domokos Z and Czumbel LM and Kói T and Hegyi P and Hermann P and Borbély J and Cavalcante BGN and Németh O | 2023 |
| [56] Teledentistry: a systematic review of clinical outcomes, utilization and costs | United States | Critically low | Daniel SJ and Wu L and Kumar S | 2013 |
| [57] Teledentistry: a systematic review of the literature | England | Critically low | Mariño R and Ghanim A | 2013 |
| [58] Telemedicine in the diagnosis and management of temporomandibular disorders: a systematic review conducted according to PRISMA guidelines and the Cochrane Handbook for Systematic Reviews of Interventions | United Kingdom | Moderate | Abdul NS and Kumari M and Shenoy M and Shivakumar GC and Herford AS and Cicciù M and Minervini G | 2023 |
| [59] The accuracy of virtual setup in simulating treatment outcomes in orthodontic practice: a systematic review | Switzerland | Low | Sereewisai B and Chintavalakorn R and Santiwong P and Nakornnoi T and Neoh SP and Sipiyaruk K | 2023 |
| [60] The feasibility of telehealth in the monitoring of head and neck cancer patients: a systematic review on remote technology, user adherence, user satisfaction, and quality of life | Germany | High | da Silva HEC and Santos GNM and Ferreira Leite A and Mesquita CRM and de Souza Figueiredo PT and Miron Stefani C and de Santos Melo N | 2022 |
| [61] The influence of mobile applications and social media-based interventions in producing behavior change among orthodontic patients: A systematic review and meta-analysis | United States | High | Al-Moghrabi D and Alkadhimi A and Tsichlaki A and Pandis N and Fleming OS | 2022 |
| [62] The scope of dental education during COVID-19 pandemic: A systematic review | United States | High | Santos GNM and da Silva HEC and Leite AF and Mesquita CRM and Figueiredo PTS and Stefani CM and Melo NS | 2021 |
| [63] The use of mobile devices in oculoplastic and oral and maxillofacial surgery: A systematic review | United States | Critically low | Zaheer HA and Jabir AR and Yang K and Othman S and Kaleem SZ and McKinnon BJ | 2022 |
| [64] Use of Virtual Reality for the Management of Anxiety and Pain in Dental Treatments: Systematic Review and Meta-Analysis | Switzerland | Critically low | López-Valverde N and Muriel-Fernández J and López-Valverde A and Valero-Juan LF and Ramírez JM and Flores-Fraile J and Herrero-Payo J and Blanco-Antona LA and Macedo-de-Sousa B and Bravo M | 2020 |
| [65] Using teledentistry in clinical practice as an enabler to improve access to clinical care: A qualitative systematic review | England | Critically low | Irving M and Stewart R and Spallek H and Blinkhorn A | 2018 |
| [66] Using Virtual Technology for Fear of Medical Procedures: A Systematic Review of the Effectiveness of Virtual Reality-Based Interventions | England | Critically low | Kılıç A and Brown A and Aras I and Hui R and Hare J and Hughes LD and McCracken LM | 2021 |
| [67] Watch and learn? A systematic review comparing oral health educational videos with written patient information aimed at parents/carers or children | England | Critically low | Loy F and Underwood B and Stevens C | 2021 |
| [68] Where Is the Artificial Intelligence Applied in Dentistry? Systematic Review and Literature Analysis | Switzerland | Critically low | Thurzo A and Urbanová W and Novák B and Czako L and Siebert T and Stano P and Mareková S and Fountoulaki G and Kosnáčová H and Varga I | 2022 |
