# Supplementary material for: Teledentistry Applied to Health and Education Outcomes: Evidence Gap Map
Source: J Med Internet Res. 2024 Nov 27;26:e60590. doi: 10.2196/60590 (PMC11635335; doi:10.2196/60590)
Supplement: Multimedia Appendix 3 [file jmir_v26i1e60590_app3.docx]

**APPENDIX 3 - EXCLUDED AFTER FULL TEXT AND REASON FOR EXCLUSION**

| **Reference** | **Reason of Exclusion** | **Link or DOI** |
| --- | --- | --- |
| [1] A Meta-analysis and Systematic Review Comparing the Effectiveness of Traditional and Virtual Surgical Planning for Orthognathic Surgery: Based on Randomized Clinical Trials. | Research outside the scope of Teledentistry | https://pubmed.ncbi.nlm.nih.gov/33031773/ |
| [2] Electronic Data Capture Versus Conventional Data Collection Methods in Clinical Pain Studies: Systematic Review and Meta-Analysis. | Research outside the scope of Teledentistry | https://pubmed.ncbi.nlm.nih.gov/32348259/ |
| [3] Efetividade de aplicativos móveis para mudanças comportamentais em saúde: revisão sistemática | Research outside the scope of Teledentistry | http://www.revenf.bvs.br/scielo.php?script=sci_arttext&pid=S1517-38522020000100402 |
| [4] Physicians' perceptions of clinical supervision and educational support via videoconference: a systematic review. | Research outside the scope of Teledentistry | https://pubmed.ncbi.nlm.nih.gov/24906649/ |
| [5] School dental screening programmes for oral health. | Research outside the scope of Teledentistry | https://pubmed.ncbi.nlm.nih.gov/31425627/ |
| [6] Four-Dimensional Superimposition Techniques to Compose Dental Dynamic Virtual Patients: A Systematic Review. | Research outside the scope of Teledentistry | https://pubmed.ncbi.nlm.nih.gov/36662080/ |
| [7] Advancement of teledentistry at the university of rochester’s eastman institute for oral health | Research outside the scope of Teledentistry | https://www.embase.com/search/results?subaction=viewrecord&id=L626876363&from=export U2 - L626876363 |
| [8] Forensic odontology with digital technologies: A systematic review. | Research outside the scope of Teledentistry | https://pubmed.ncbi.nlm.nih.gov/32658766/ |
| [9] Natural language processing for clinical notes in dentistry: A systematic review. | Research outside the scope of Teledentistry | https://pubmed.ncbi.nlm.nih.gov/31334142/ |
| [10] A mixed methods systematic review of the effects of patient online self-diagnosing in the 'smart-phone society' on the healthcare professional-patient relationship and medical authority. | Research outside the scope of Teledentistry | https://pubmed.ncbi.nlm.nih.gov/33023577/ |
| [11] Are remote clinical assessments a feasible and acceptable method of assessment? A systematic review. | Research outside the scope of Teledentistry | https://pubmed.ncbi.nlm.nih.gov/34665073/ |
| [12] Gamification for health promotion: systematic review of behaviour change techniques in smartphone apps. | Research outside the scope of Teledentistry | https://pubmed.ncbi.nlm.nih.gov/27707829/ |
| [13] Deep learning for caries detection: A systematic review. | Research outside the scope of Teledentistry | https://pubmed.ncbi.nlm.nih.gov/35367318/ |
| [14] Diagnostic accuracy and measurement sensitivity of digital models for orthodontic purposes: A systematic review. | Research outside the scope of Teledentistry | https://pubmed.ncbi.nlm.nih.gov/26827972/ |
| [15] Intraoperative augmented reality with heads-up displays in maxillofacial surgery: a systematic review of the literature and a classification of relevant technologies. | Research outside the scope of Teledentistry | https://pubmed.ncbi.nlm.nih.gov/30316662/ |
| [16] The WHO Health Promoting School framework for improving the health and well-being of students and their academic achievement. | Research outside the scope of Teledentistry | https://pubmed.ncbi.nlm.nih.gov/24737131/ |
| [17] Educational strategies for teaching evidence-based practice to undergraduate health students: systematic review. | Research outside the scope of Teledentistry | https://pubmed.ncbi.nlm.nih.gov/27649902/ |
| [18] Oral health educational interventions for nursing home staff and residents. | Research outside the scope of Teledentistry | https://pubmed.ncbi.nlm.nih.gov/27689868/ |
| [19] OPEN-SOURCE SOFTWARE IN DENTISTRY: A SYSTEMATIC REVIEW. | Research outside the scope of Teledentistry | https://pubmed.ncbi.nlm.nih.gov/28857016/ |
| [20] Current state of the art in the use of augmented reality in dentistry: a systematic review of the literature. | Research outside the scope of Teledentistry | https://pubmed.ncbi.nlm.nih.gov/31286904/ |
| [21] Predicting Factors on Modeling Health Behavior: A Systematic Review. | Research outside the scope of Teledentistry | https://pubmed.ncbi.nlm.nih.gov/33888188/ |
| [22] Systematic literature review of digital three-dimensional superimposition techniques to create virtual dental patients. | Research outside the scope of Teledentistry | https://pubmed.ncbi.nlm.nih.gov/25830393/ |
| [23] Systematic Review and Meta-analysis of Virtual Reality in Pediatrics: Effects on Pain and Anxiety. | Research outside the scope of Teledentistry | https://pubmed.ncbi.nlm.nih.gov/31136330/ |
| [24] A systematic review of 3D scanners and computer assisted analyzes of bite marks: searching for improved analysis methods during the Covid-19 pandemic. | Research outside the scope of Teledentistry | https://pubmed.ncbi.nlm.nih.gov/34302214/ |
| [25] Accuracy of digital technologies for the scanning of facial, skeletal, and intraoral tissues: A systematic review. | Research outside the scope of Teledentistry | https://pubmed.ncbi.nlm.nih.gov/30017156/ |
| [26] Accuracy of Virtual Static Articulation: A Systematic Review. | Research outside the scope of Teledentistry | https://pubmed.ncbi.nlm.nih.gov/35349610/ |
| [27] Does watching an informative video reduce the anxiety in patients undergoing third molar surgery: a systematic review of randomized controlled trials. | Research outside the scope of Teledentistry | https://pubmed.ncbi.nlm.nih.gov/36525143/ |
| [28] Remote provision of breastfeeding support and education: Systematic review and meta-analysis. | Research outside the scope of Teledentistry | https://pubmed.ncbi.nlm.nih.gov/34964542/ |
| [29] Augmented and Virtual Reality in Anatomical Education - A Systematic Review. | Research outside the scope of Teledentistry | https://pubmed.ncbi.nlm.nih.gov/32488637/ |
| [30] TELEMEDICINE : COVID -19 PANDEMIC AND THE RISE OF  THE VIRTUAL CARE IN NEPHROLOGY IN SPAIN | Research outside the scope of Teledentistry | https://pubmed.ncbi.nlm.nih.gov/36662080/ |
| [31] Temporomandibular disorders and orofacial neuropathic pain in children and adolescents: a systematic review. | Research outside the scope of Teledentistry | https://pubmed.ncbi.nlm.nih.gov/37143419/ |
| [32] Telemedicine : COVID-19 pandemic and the rise of the virtual care in nephrology in Spain | Research outside the scope of Teledentistry | https://pesquisa.bvsalud.org/global-literature-on-novel-coronavirus-2019-ncov/resource/pt/covidwho-1402466 |
| [33] Implementation of blockchain technology across different domains of dentistry: a systematic review. | Research outside the scope of Teledentistry | https://pubmed.ncbi.nlm.nih.gov/37868487/ |
| [34] Big Data and Digitalization in Dentistry: A Systematic Review of the Ethical Issues. | ICT with no clinical or educational applications | https://pubmed.ncbi.nlm.nih.gov/32268509/ |
| [35] Augmented and virtual reality in dental medicine: A systematic review. | ICT with no clinical or educational applications | https://pubmed.ncbi.nlm.nih.gov/31003184/ |
| [36] Integration of oral health into primary health care: A systematic review. | ICT with no clinical or educational applications | https://pubmed.ncbi.nlm.nih.gov/31334142/ |
| [37] Teledentistry and the unified health system: An important tool for the resumption of primary health care in the context of the covid-19 pandemic | Neither systematic review or meta-analysis | https://www.embase.com/search/results?subaction=viewrecord&id=L2004978889&from=export U2 - L2004978889 |
| [38] Teledentistry: Development and Management of Telehealth in Dentistry | Neither systematic review or meta-analysis | https://www.embase.com/search/results?subaction=viewrecord&id=L2016505274&from=export U2 - L2016505274 |
| [39] Panorama situacional da Teleodontologia no mundo: uma revisão integrativa | Neither systematic review or meta-analysis | https://revabeno.emnuvens.com.br/revabeno/article/view/455/442 |
| [40] Mobile Apps for Dental Caries Prevention: Systematic Search and Quality Evaluation. | Neither systematic review or meta-analysis | https://pubmed.ncbi.nlm.nih.gov/33439141/ |
| [41] The evolution of the teledentistry landscape in Australia: A scoping review. | Neither systematic review or meta-analysis | https://pubmed.ncbi.nlm.nih.gov/35567780/ |
| [42] The use of teledentistry in facilitating oral health for older adults: A scoping review. | Neither systematic review or meta-analysis | https://pubmed.ncbi.nlm.nih.gov/34521539/ |
| [43] To what extent is telehealth reported to be incorporated into undergraduate and postgraduate allied health curricula: A scoping review. | Neither systematic review or meta-analysis | https://pubmed.ncbi.nlm.nih.gov/34411171/ |
| [44] Retraction: López-Valverde, N.; et al. Use of Virtual Reality for the Management of Anxiety and Pain in Dental Treatments: Systematic Review and Meta-Analysis. J. Clin. Med. 2020, 9, 1025. | Neither systematic review or meta-analysis | https://pubmed.ncbi.nlm.nih.gov/32731319/ |
| [45] The role of teledentistry in oral cancer patients during the COVID-19 pandemic: an integrative literature review | Neither systematic review or meta-analysis | https://www.embase.com/search/results?subaction=viewrecord&id=L2013052282&from=export U2 - L2013052282 |
| [46] Scoping review of artificial intelligence and immersive digital tools in dental education. | Neither systematic review or meta-analysis | https://pubmed.ncbi.nlm.nih.gov/34962645/ |
| [47] Is the answer to reducing early childhood caries in your pocket? | Neither systematic review or meta-analysis | https://pubmed.ncbi.nlm.nih.gov/37582973/ |
| [48] Accuracy and effectiveness of teledentistry: a systematic review of systematic reviews. | Neither systematic review or meta-analysis | https://pubmed.ncbi.nlm.nih.gov/35804195/ |
| [49] Does using mobile applications and social media-based interventions induce beneficial behavioral changes among orthodontic patients? | Neither systematic review or meta-analysis | https://pubmed.ncbi.nlm.nih.gov/36882496/ |
| [50] Provision of Endodontic Treatment in Dentistry amid COVID-19: A Systematic Review and Clinical Recommendations. | Clinical approach without the use of ICT | https://pubmed.ncbi.nlm.nih.gov/34901279/ |
